# Why is DNA methylation of *Igf2 CpG* island shore affected during ageing?

**DOI:** 10.18632/aging.100473

**Published:** 2012-01-08

**Authors:** Paola Caiafa, Michele Zampieri

**Affiliations:** Department of Cellular Biotechnologies and Hematology, Faculty of Pharmacy and Medicine, Sapienza University of Rome, 00161 Rome, Italy

In post-genomic era attention has been focused on the characterization of the human DNA “methylome” at single nucleotide resolution. It is a particularly difficult goal to achieve as methylation profiling is different in tissues and cells of the same individual. 5- Methylcytosine is considered the “fifth base” of DNA as it introduces an epigenetic code on genome which is defined during development. Nutrition, environment and style of life have an impact on the “methylome” and the introduction of anomalous methyl groups in sequences that have to be maintained unmethylated and/or demethylation of chromatin regions which are usually methylated drive the biological events associated with pathologies. The same anomalies in methylation patterns occur with age so an in-depth knowledge of these changes will help us to individuate what leads to the passage from successful ageing to one in which there is the onset of disease [1]. Data from Franceschi's group are focused on ageing and the cohorts of individuals are selected in order to establish whether variability in DNA methylation patterns is to ascribe to age of individuals rather than to their geographical dimension. Notably, in this study monozygotic and dizygotic twins of ages ranging from 22 to 97 years are also enrolled.

Four regions, located at the *Igf2/H19* imprinted locus, were analyzed including the CpG island present in differentially methylated region 2 (DMR2) and the nearby 5’ shore, two fragments never investigated up to now. Authors conclude that the shore is the one in which the scatter in methylation variability during ageing is more evident.

Shore regions, which localize in proximity of CpG islands at 200-2000 kb away from them, are of particular interest as involved in gene expression regulation through the DMRs often located within them [2].

A long-range looping interaction is involved in the control of imprinting at maternally inherited allele at the *Igf2/H19* gene locus. An interesting hypothesis for susceptibility to epimutation of this shore region in aged individuals is that its position inside the loop is topologically responsible for its availability to change the methylation profile. Although the higher order chromatin organization at *Igf2/H19* locus is well assessed in mice, less information is available in humans.

Anyway, the analysis of data about chromatin structure from the ENCODE consortium, accessible at the UCSC Genome Browser (http://genome.ucsc.edu/) [3, 4], indicates a distinctive chromatin feature of this shore region which corresponds to an open, DNAaseI hypersensitive chromatin domain characterized by an early S-phase replication.

As chromatin structure affects the susceptibility to DNA damage, the chromatin shape of the shore could be the reason for its susceptibility to epimutation. An important link was found between DNA damage, homology directed repair and DNA methylation [5], suggesting that DNA-methyltransferase 1(DNMT1), which is recruited to repair sites following double-strand DNA breaks [6], marks the repaired segments superimposing novel methylation profile.

Another interesting hypothesis rises from the early replication timing of this shore. The maintenance of DNA methylation patterns during DNA replication requires the function of DNMT1 which is brought to the replication fork through a direct interaction with Proliferating Cell Nuclear Antigen protein (PCNA). Interestingly, DNMT1 forms a complex at replication foci only during late stages of S-phase when methylated DNA is replicated. Thus, the controlled recruitment of DNMT1 into replication forks is an important component in the perpetuation of DNA methylation patterns and S-phase abnormalities could affect methylation patterns by altering the replication timing of sequences. The biological event able to disrupt DNMT1 function in S phase could be poly(ADP-ribosyl)ation. It has been found that competitive inhibition of PARPs leads to hyperexpression of DNMT1 in G1/early S phase, increases the amount of DNMT1 that co-immunoprecipitates with PCNA in this phase and causes DNA hypermethylation [7]. Interestingly, reduced levels of poly(ADP-ribosyl)ation enzymes PARP1 and 2 and poly(ADP-ribosyl)ation activity have been associated with ageing [8, 9].

In conclusion, changes of methylation profile at *Igf2 CpG* island shore observed during ageing could be due to the loss of local epigenetic control of this region. The understanding of molecular mechanism(s) involved in the protection of the methylation pattern of this shore could provide important information about the participation of epigenetics in ageing and disease.
